# Stability of Sodium Pentobarbital 5w/v% Aqueous Solution During Short‐Term Chilled and Ambient‐Temperature Storage

**DOI:** 10.1002/vms3.70066

**Published:** 2024-10-26

**Authors:** Masakazu Dohi, Kayoko Ueda, Yoshinori Yamagiwa, Atsuko Yamashita, Yuji Sakamoto

**Affiliations:** ^1^ Central Research Laboratories, Research & Development Division Senju Pharmaceutical Co. Ltd. Kobe Hyogo Japan

**Keywords:** anaesthetics agents, chemical analysis, euthanasia, pentobarbital

## Abstract

The COVID‐19 pandemic made it difficult to obtain pharmaceutical‐grade injectable anaesthetics used in animal experiments in Japan. To address this problem, it is worth the effort to evaluate whether the use of non‐pharmaceutical‐grade sodium pentobarbital (PBNa) water solution can be an alternative one from the perspective of stability, physicochemical properties, bioavailability and so on. In this article, as a first step, we examined the stability of PBNa 5w/v% aqueous solution (PBNa 5% AS). PBNa 5% AS sample was prepared simply by dissolving non‐pharmaceutical‐grade PBNa into water and was stored at 25°C and 5°C for 15 days. Visual appearance, pH, osmolality and assay (contents of PBNa and related substances) were tested. The stability test results indicated that there was no profound change in these test items of the PBNa 5% AS under either storage condition, suggesting that the formulation was sufficiently stable in such a short term. The only observable change was an increase of an impurity under 25°C storage; however, it was at a negligible level. Moreover, as the pH of the formulation remained <10.0 throughout the storage period in both conditions, the irritant level of the pH is considered to be acceptable. Therefore, from the viewpoint of the stability and physicochemical properties, PBNa 5% AS can be an alternative injectable anaesthetic when access to pharmaceutical‐grade anaesthetics is limited, as long as used in short‐term from its preparation.

## Graphical abstract

Sodium pentobarbital (PBNa) 5w/v% aqueous solution was able to be prepared simply by dissolving non‐pharmaceutical‐grade sodium pentobarbital into water. This study clarified that the prepared solution was stable for 15 days under both 25°C and 5°C and had allowable physicochemical properties for animals. Therefore, the PBNa 5w/v% aqueous solution is an alternative injectable anaesthetic for experimental animals when access to pharmaceutical‐grade anaesthetics is limited.

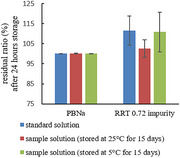

## 1 Introduction

Barbiturates are compounds exerting anxiolytic, hypnotic and anticonvulsant effects, and they are widely used as anaesthetics in animal experiments. In general, anaesthetics used in academic and commercial animal experiments, including barbiturates, should be of pharmaceutical‐grade, as instructed in the ILAR Guide (Institute for Laboratory Animal Research 2011). In compliance with the principle, academic and corporate researchers engaged in laboratory animal experiments generally use commercially available pharmaceutical‐grade anaesthetics. However, in Japan, the COVID‐19 pandemic has limited access to pharmaceutical‐grade anaesthetics including barbiturates, because of their prioritized use for medical purposes. The resultant shortage of pharmaceutical‐grade barbiturates in laboratories has been forcing researchers to use non‐pharmaceutical‐grade barbiturates or other alternative anaesthetics in their animal experiments. In accordance with the ILAR Guide, when performing an animal experiment with non‐pharmaceutical‐grade barbiturate, the physicochemical properties and stability of its dosage form should be cared to guarantee its effectiveness. Therefore, it is supposed to be necessary to establish appropriate internal standard operating procedures to define the composition and preparation method of the injectable formulations used. The situation is somewhat more complicated when focusing on Japanese case regarding pentobarbital or its sodium salt, which is a barbiturate that is typically used in animal experiments. This is because a key distributor of its pharmaceutical‐grade injectable formulation terminated its commercial supply in Japan before the COVID‐19 spread worldwide. Before the COVID‐19 turmoil arose, researchers could manage to address this vendor problem by using other barbiturates (pharmaceutical‐grade) than pentobarbital. However, the current pandemic has made it difficult even to get these alternatives.

Regarding adequate usage of non‐pharmaceutical‐grade pentobarbital, for example, the University of Illinois Urbana‐Champaign, the University of Maryland Baltimore and Priest et al. have proposed methods of preparation and compositions of its injectable formulations, each of which includes ethanol and propylene glycol as components (University of Illinois Urbana‐Champaign 2014, 2021; Priest and Geisbuhler 2015). Priest and Geisbuhler (2015) also showed that their formulation was so stable at room temperature that the loss of pentobarbital did not exceed 0.5% per year. Although ethanol and propylene glycol reasonably contribute to the suppression of precipitation and chemical degradation, they are conceivably harmful to experimental animals to a certain extent (Priest and Geisbuhler 2015). In addition, these preparation methods are time‐consuming.

It is typically not required that drugs have a long shelf life (e.g. 3 years) with regard to anaesthetic formulations used in animal experiments, thus replacing the abovementioned complex formulations with simpler ones may be preferable from the viewpoint of experimental animal welfare and ease of preparation at a negligible expense of stability. As the extreme of a simple formulation of pentobarbital, a sodium pentobarbital 5w/v% aqueous solution (PBNa 5% AS) is worth investigating. In this study, we performed a stability test on PBNa 5% AS stored at 25°C and 5°C for 15 days to evaluate its potential usability as an alternative anaesthetic formulation of pentobarbital.

## 2 Materials and Methods

### 2.1 Materials and Preparation and Storage of Aqueous Solution

Powdered crystalline PBNa was purchased from Nacalai Tesque Inc. (Kyoto, Japan), at a purity specification of ≥95%. Immediately after removing the seal from the newly purchased bottle, about 9.0 g of PBNa was accurately weighed using MSA225P‐000‐DI Balance (Sartorius Japan K. K., Tokyo, Japan) and placed in a glass beaker without drying. Approximately 180 mL ultrapure Milli‐Q water was added, followed by stirring for approximately 5 min at room temperature to give a completely dissolved aqueous solution of PBNa (PBNa 5% AS). The homogeneous solution was divided into several aliquots (5 mL, each), which were then placed in glass bottles with lids tight. The glass bottles were covered with aluminium foil to protect the content from light exposure and were placed in two thermostatic chambers set at 25°C and 5°C, respectively. The samples thus stored in both the chambers were subjected to a stability study as specimens. A test was performed in three iterations for each test item at each sampling point, that is at 0, 1, 5 and 15 days of storage.

### 2.2 Stability Test

Visual appearance, pH, osmolality and assay (content of PBNa and related substances) were tested at each sampling point at 25°C and 5°C. Tests on Day 0 (before samples were placed in the chambers) were performed only for the 25°C samples as no treatment effects would have occurred at this point.

Visual appearance was examined according to [Description] of the General Notices of the Japanese Pharmacopeia 18 Edition (JP18th), that is by pouring the sample into a clear glass test tube, followed by visual check of its colour and clarity. pH was tested using an F‐72 pH meter (HORIBA Advanced Techno Co. Ltd., Kyoto, Japan) according to [pH Determination] of the General Tests 〈2.54〉 of the JP18th. Osmolality was tested using an A_2_O Osmometer (Advanced Instruments Ltd., Norwood, MA, USA) according to [Osmolarity Determination] of the General Tests 〈2.47〉 of the JP18th, which makes use of the extent of the freezing‐point depression. Assay (contents of PBNa and related substances) was performed using prominence, a high‐performance liquid chromatograph (HPLC; SHIMADZU CORPORATION, Kyoto, Japan), with minor modifications of the method of Morley et al. (Morey and Elrod 1997). That is, to 1 mL of the stored sample, the mobile phase (see Table 1 for its composition) was added to make exactly 100 mL. To 1 mL of this solution, the mobile phase was added to exactly 10 mL to give a sample solution. Separately, about 10 mg of PBNa previously dried at 105°C for about 2 h was accurately weighed and dissolved in the mobile phase to produce exactly 50 mL. To 2.5 mL of this solution, the mobile phase was added to make exactly 10 mL to give the standard solution, which was prepared fresh for each analysis run. A volume of 50 µL of the sample and standard solutions were injected to the HPLC, and the test was performed according to the operating conditions shown in Table 1. Each peak area was determined by the automatic integration method, and the contents of PBNa in the sample solution and that of a related substance were calculated by the formulae below. For related substances, we calculated only the contents of peaks that were observed to obviously increase during the storage period (by visual inspection of the chromatograms). On performing the assay, the UV spectra were also obtained by cutting out the 3D spectra at the elution time of PBNa with a photodiode array (PDA; wavelength: 190–600 nm) for the standard solution, sample solution of the sample stored at 25°C for 15 days, and that of the sample stored at 5°C for 15 days. All examinations were conducted in the Laboratory of Senju Pharmaceutical Co. Ltd., which is managed under Japanese investigational drug Good Manufacturing Practice (GMP)‐compliant organization:

$$\mathrm{Content}\;\left(\%\right)\;\mathrm{of}\;\mathrm{PBNa}=10\;\times \;W_{S}\;\times \;\left(A_{T}/A_{S}\right)$$

$$\mathrm{Content}\left(\%\right)\mathrm{of}\;a\;\mathrm{related}\;\mathrm{substance}=10\;\times \;W_{S}\;\times \;\left(A_{T}\left(\mathrm{rs}\right)/A_{S}\right),$$

where *W*_S_ is the amount (mg) of PBNa taken as reference standard, *A*_T_ is the peak area of PBNa in sample solution, *A*_T_(rs) is the peak area of related substance in sample solution, and *A*_S_ is the peak area of PBNa in standard solution.

**TABLE 1:** Operating conditions of the high‐performance liquid chromatograph (HPLC) method used for the assay (contents of sodium pentobarbital [PBNa] and related substances).

Detector:	Ultraviolet absorption photometer (wavelength: 214 nm) Photodiode array (PDA) (wavelength: 190–600 nm)
Column:	A stainless steel column 4.6 mm in inside diameter and 15 cm in length, packed with octadecylsilanized silica gel for liquid chromatography (particle diameter: 5 µm, pore size: 100 Å; InertSustain C18, GL Sciences)
Column temperature:	A constant temperature of about 25°C
Sample cooler temperature:	A constant temperature of about 25°C
Mobile phase:	A calcium phosphate buffer (pH 3.5, 0.14 w/v%)/acetonitrile (72:28)
Flow rate:	1.0 mL/min
Flowing of mobile phase:	Isocratic
Time span of measurement:	45 min

### 2.3 Assay (Content of PBNa) on Filtrate

To deny the possibility that invisible tiny PBNa precipitates decreased the amount of dissolved PBNa to an unacceptable level, filtration of the stored samples and the assay (content of PBNa) on the filtrate was performed as follows: 5 mL samples stored at 25°C and 5°C for 16 days were filtrated through 0.45‐µm filters (autoclaved olefin‐type polymer, GL Sciences Inc., Tokyo, Japan). In order to saturate the adsorption of PBNa onto the filters, the first 5 mL of the samples was passed through the filters and were discarded. Subsequently, the remaining samples were filtered again in the same way and were collected. The assay (content of PBNa) was performed on the resulting filtrates, according to the aforementioned method.

### 2.4 Powder X‐Ray Diffraction (pXRD)

In the filtrates obtained by performing the assay on the filtrate, white solids were observed to precipitate 1 day after filtration. On the precipitates generated from the filtrates of the samples stored at 25°C (Sample A) and 5°C (Sample B) along with the commercially available PBNa (Sample C), we performed pXRD using a desktop X‐ray diffractometer MiniFlex 600 (Rigaku Corporation, Tokyo, Japan) after pretreatment. Sample C was the same lot as used for the preparation of the PBNa 5% AS. For Samples A and B, we dried the filtrates that had been left in glass beakers without a lid on the laboratory bench for 67 days from filtration in vacuum at 42°C for 1 h to evaporate the residual water almost completely. The samples were then ground using a mortar and pestle to prepare specimens for pXRD. Sample C was only ground in a similar way without drying, and pXRD was performed on it. The pXRD patterns of thus pretreated Samples A, B and C were corrected using Cu *K*_α_ radiation (1.54186 Å) with an anode at a voltage of 40 kV and a current of 15 mA.

### 2.5 Concise Validation of Assay (Contents of PBNa and Related Substances)

Because the analytical method for the assay (contents of PBNa and related substances) was not validated, we performed a concise analytical method validation and evaluated only two characteristics, that is, the linearity and stability of the analyte solutions. For linearity, solutions with 80%, 90%, 100%, 110% and 120% concentrations of the standard solution were prepared and analysed. From their responses and concentrations, *R* ^2^ was calculated. With regard to analyte solution stability, the standard and sample solutions contained in glass vials that were prepared for the measurement of the 15‐day sampling point at 25°C and 5°C were kept stored in a sample cooler at 25°C. Twenty‐four hours after the first analysis, the analyte solutions were measured again (three iterations). The residual ratios (%) of PBNa and related substances were calculated using the following formula:

$$\mathrm{Residual}\;\mathrm{ratio}\left(\%\right)=A_{s}/A_{f}\;\times \;100$$

where *A*_f_ is the peak area of PBNa or related substance of the first analysis, and *A*_s_ is the peak area of PBNa or related substance of the second analysis.

## 3 Results

### 3.1 Stability

**FIGURE 1:**
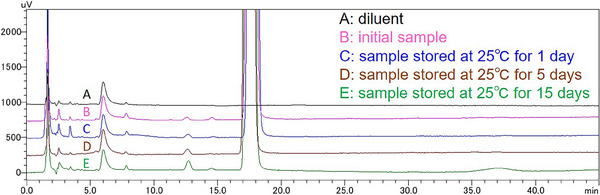
Magnified chromatograms obtained by the assay (contents of PBNa and related substances) on the samples stored at 25°C. The chromatograms shown are those of the diluent (A), and the sample stored at 25°C for 0 day (B, initial sample), 1 day (C), 5 days (D) and 15 days (E). The peak eluted about at 12.7 min (relative retention time [RRT] 0.72 impurity) is the only peak that was observed to obviously increase during storage. PBNa, sodium pentobarbital.

**FIGURE 2:**
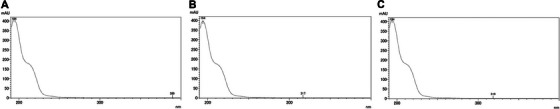
UV spectra with PDA of the standard solution (A), the sample solution of the sample stored at 25°C for 15 days (B) and the sample solutions of the sample stored at 5°C for 15 days (C).

The results of the stability tests are shown in Table 2, except for those of the PDA UV spectra. With regard to related substances, visual inspection of magnified chromatograms clarified that at 25°C, there was only one peak which obviously increased during storage at 25°C (Figure 1), whereas at 5°C, there was no peak observed to increase. The retention time and relative retention time (RRT) against PBNa of the peak that increased at 25°C were approximately 12.7 min and 0.72, respectively. Therefore, only the content of this RRT 0.72 impurity was calculated as the result of the assay of related substances. The UV spectra at the retention time of PBNa obtained using PDA are compared in Figure 2.

**TABLE 2:** Results of stability test on pentobarbital 5w/v% aqueous solution (PBNa 5% AS) samples stored at 25°C (A) and 5°C (B) for 15 days.

(A)					
Item	Iterations	Day 0 (initial)	Day 1	Day 5	Day 15
Visual appearance^a^	1st	*	*	*	*
	2nd	*	*	*	*
	3rd	*	*	*	*
pH	1st	9.87	9.77	9.79	9.83
	2nd	9.90	9.78	9.77	9.87
	3rd	9.89	9.71	9.79	9.91
	Mean	9.9	9.8	9.8	9.9
Osmolality^b^	1st	1.3 (379)	1.3 (379)	1.3 (382)	1.3 (380)
	2nd	1.3 (379)	1.3 (380)	1.3 (383)	1.3 (381)
	3rd	1.3 (379)	1.3 (380)	1.3 (383)	1.3 (381)
	Mean	1.3	1.3	1.3	1.3
Content (%) of PBNa	1st	97.5	96.8	99.1	99.4
	2nd	100.5	100.9	98.1	102.2
	3rd	102.9	101.4	98.9	102.4
	Mean	100.3	99.7	98.7	101.3
Content (%) of	1st	0.021	0.021	0.036	0.067
RRT 0.72 related	2nd	0.017	0.021	0.036	0.070
substance	3rd	0.017	0.019	0.034	0.071
	Mean	0.018	0.020	0.035	0.069

(B)					
Item	Iterations	Day 0 (initial)	Day 1	Day 5	Day 15
Visual appearance^a^	1st	—	*	*	*
	2nd	—	*	*	*
	3rd	—	*	*	*
pH	1st	—	9.79	9.79	9.94
	2nd	—	9.78	9.79	9.92
	3rd	—	9.80	9.80	9.91
	Mean	—	9.8	9.8	9.9
Osmolality^b^	1st	—	1.3 (379)	1.3 (383)	1.3 (381)
	2nd	—	1.3 (380)	1.3 (381)	1.3 (381)
	3rd	—	1.3 (379)	1.3 (382)	1.3 (380)
	Mean	—	1.3	1.3	1.3
Content (%) of PBNa	1st	—	95.9	97.4	101.6
	2nd	—	99.0	98.0	101.6
	3rd	—	101.0	98.6	101.9
	Mean	—	98.6	98.0	101.7
Content (%) of	1st	—	0.018	0.017	0.019
RRT 0.72 related	2nd	—	0.019	0.017	0.021
Substance	3rd	—	0.018	0.020	0.020
	Mean	—	0.018	0.018	0.020

Abbreviation: RRT, relative retention time.

^a^ The asterisk (*) indicates that the stored sample was a colourless clear solution, and no solids were observed.

^b^ For osmolality, the values outside parentheses and those inside them indicate the osmotic pressure ratio and osmotic pressure, respectively.

### 3.2 Filtrate Assay

The PBNa content in the filtrates of the samples stored for 16 days at 25°C and 5°C was 100.7% and 99.8%, respectively. These values were almost the same as those of unfiltered samples stored for 15 days (Table 2).

After completion of the analyses of the filtrates, we left the residual filtrates being in glass beakers without a lid on the laboratory bench for over 2 months. One day after filtration, considerable amounts of white solid precipitate were observed in both filtrates of samples stored at 25°C and 5°C. In contrast, in the unfiltered samples stored at 25°C and 5°C in the chambers, no precipitate was observed by visual inspection for at least 5 months.

### 3.3 Powder X‐Ray Diffraction

**FIGURE 3:**
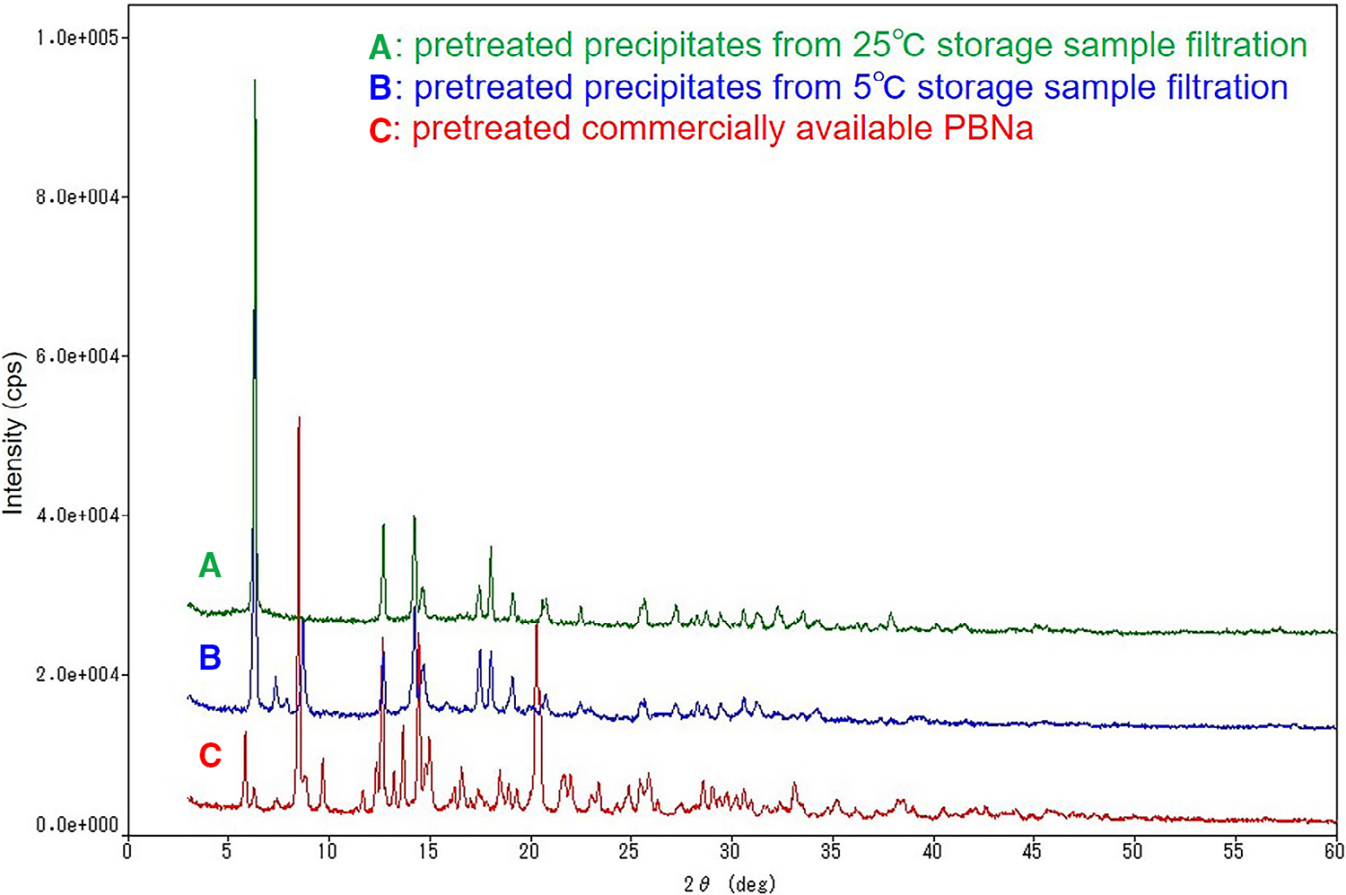
Powder X‐ray diffraction (pXRD) patterns of the pretreated sample (A); the precipitates generated from the filtrate of the sample stored at 25°C (A), the pretreated sample (B); the precipitates generated from the filtrate of the sample stored at 5°C (B) and (C); the commercially available PBNa (C). The patterns of A and B are much the same as the pattern of a pentobarbital free form. PBNa, sodium pentobarbital.

The obtained pXRD patterns of the pretreated Samples A, B and C are shown in Figure 3 (Sample A: precipitates generated from the filtrate of the sample stored at 25°C; Sample B: precipitates generated from the filtrate of the sample stored at 5°C; Sample C: commercially available PBNa). The diffraction patterns of the pretreated Samples A and B closely resembled each other, whereas pretreated Sample C exhibited a different pattern from those of A and B.

### 3.4 Concise Validation

**FIGURE 4:**
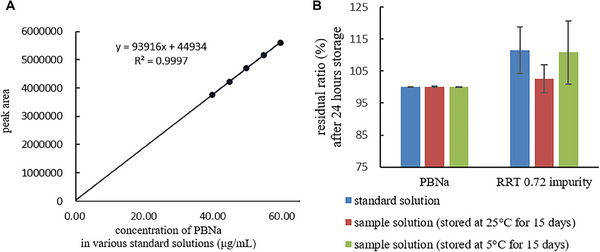
Result of the linearity evaluation for the assay of content of PBNa (A) and the stability of analyte solutions (B). Error bars indicate the sample standard deviation of three iterations. PBNa, sodium pentobarbital.

The evaluation of linearity exhibited an *R* ^2^ as high as 0.9997, with the *y*‐intercept being near zero (Figure 4A). As for the stability of the analyte solutions in the sample cooler, the result is shown in Figure 4B. Regarding related substances, only RRT 0.72 impurity was subjected to the analysis, for the same reason explained above.

## 4 Discussion

We investigated the stability of a simple anaesthetic formulation of PBNa 5% AS during 15 days storage at 25°C and 5°C. Among the test items evaluated in the two conditions, the only observable change was the increase of the RRT 0.72 impurity at 25°C (Table 2). The structure of this impurity was not elucidated; however, one possible structure is ring‐opened PBNa due to hydrolysis (Brynjelsen and Pati 2016). The increased amount of this impurity was only 0.05% after 15 days of storage at 25°C. Simple linear extrapolation suggests that the increase in the impurity at 25°C was approximately 1.2% per year of storage. However, the present extent of the impurity was not expected to be problematic for the use of the anaesthetic in animal experiments, as long as it is used about within 2 weeks from preparation. It was estimated that the relatively high degree of decomposition was attributed to the absence of ethanol and propylene glycol and the relatively low pH (Brynjelsen and Pati 2016). In the assay, there was no observable difference between the UV spectra at the retention time of PBNa obtained with PDA (Figure 2). Based on that, we speculated that the peaks consisted of almost all of PBNa throughout the 15 days storage at 25°C and 5°C. In addition, the results of the filtrate assay supported that the generation of PBNa solids in the conditions was, if at all, negligible level. Given these results, along with the results of the concise validation of the assay (contents of PBNa and related substances), we concluded that PBNa 5% AS is sufficiently stable at 25°C and 5°C for 15 days.

In contrast to these desirable results, we observed that after filtration of PBNa 5% AS, precipitation of white solids was far more likely to occur than in the unfiltered sample. Because the pXRD patterns of the treated filtrates of the samples stored at 25°C (Sample A) and 5°C (Sample B) did not match that of the commercially available PBNa (Sample C), we performed a literature search on the polymorph information on PBNa; however, no beneficial information was available. By contrast, regarding the polymorph of its free form (pentobarbital), Griesser et al. reported the existence of the four different forms, and the pattern of its form IV matches those of the treated Samples A and B well (Rossi et al. 2012). This suggests that the yielded precipitates in the current study were not the sodium salts but the free forms. The cause of the precipitation was possibly a physical stimulus, minute pH changes or eluted material from the filter; however, we have not yet identified the cause. This risk of precipitation can be reduced by performing filtration immediately before administration of the anaesthetic, although we have not investigated other types of filters that may improve this situation. In any case, users should thus visually confirm the absence of precipitates in the solution before administration.

Generally, minimizing the additives in drug formulations used for administration to animals is desirable because additives may affect their health conditions. Nevertheless, almost all pentobarbital formulations proposed so far for laboratory use contain ethanol and propylene glycol (University of Illinois Urbana‐Champaign 2014, 2021). Both alcohol agents contribute to the solution stability of barbiturates, including PBNa (Brynjelsen and Pati 2016). However, excessive intravenous injection of ethanol to mice and rats is known to induce haematuria and abnormal behaviours (Gad et al. 2016). Further, intravenous injection of propylene glycol to rats results in haemolysis (Gad et al. 2016). Given the information, the use of pentobarbital formulations without alcoholic additives such as PBNa 5% AS may help euthanize laboratory animals without the appearance of these symptoms. Separately, adjustment of pH of dosing formulations is required to relieve irritation or pain of the animals. Previous studies reported that the pH of PBNa aqueous solution could not be adjusted to <10 without the risk of precipitation (Laferriere and Pang 2020). In the present study, however, the pH of the formulation was <10.0 throughout storage for 15 days. In addition, it is important that the measured pH here is accurate, whereas the pH of solutions containing organic solvents cannot be determined accurately. Although any study including this study has not compared the adverse effects of PBNa 5% AS and the alcoholic additive‐contained solutions on animals, these facts suggest that the PBNa 5% AS prepared in the present study is more tolerable to animals than solutions containing alcoholic additives.

## 5 Conclusion

In conclusion, from the viewpoint of the stability and physicochemical properties, non‐pharmaceutical‐grade PBNa 5% AS can be considered an alternative injectable anaesthetic of pharmaceutical‐grade ones, if used within the period ensured in the current study.

## Author Contributions

**Masakazu Dohi**: conceptualization, data curation, formal analysis, funding acquisition, investigation, methodology, project administration, writing–original draft. **Kayoko Ueda**: project administration, writing–review and editing. **Yoshinori Yamagiwa**: writing–review and editing. **Atsuko Yamashita**: writing–review and editing. **Yuji Sakamoto**: writing–review and editing.

## Ethics Statement

The authors have nothing to report.

## Consent

No animals were used in this study.

## Conflicts of Interest

The authors declare no conflicts of interest.

### Peer Review

The peer review history for this article is available at https://publons.com/publon/10.1002/vms3.70066.

## Data Availability

The data that support the findings of this study are available from the corresponding author upon reasonable request.
